# Notes and News

**Published:** 1897-04-24

**Authors:** 


### Notes and News.

(Collected and Communicated.)

The treasurer of Charing Cross Hospital has received a munificent donation of £2,000 from Miss Leicester to be applied to the erection of a chapel, and also a donation of £500 from the Baroness de Hirsch to the Special Appeal Fund.

The honorary medal of the Royal College of Surgeons was presented to Lord Lister and Sir James Paget at the last meeting of the Council. Only ten persons since the foundation, in 1800, of the medal have been presented with it.

Miss L. A. Williams has given £1,000 to endow a bed at Guy's Hospital in memory of Dr. Henry Pratt, formerly a student at the hospital; and Charing Cross Hospital has received the same sum per Dr. Travers to endow a bed at that hospital.

For some time past small-pox has been epidemic on the Chinese Coast and at the ports, and has spread to Japan, where it is assuming alarming proportions. In Tokyo compulsory vaccination was ordered for all inhabitants under 50 years of age not recently vaccinated, and this system has been adopted elsewhere. So far, rigorous sanitary measures have kept the disease from spreading in Hong Kong.

We learn that the stamp to be issued in commemoration of the 60th anniversary of the Queen's reign in connection with the Prince of Wales's Hospital Fund will be ready for purchase early in May. The stamp, which is of beautiful design, is of two values, 1s. and 2s. 6d. respectively. Intending purchasers should order soon from some newsagent, bookseller, or stamp dealer, as the number issued is to be strictly limited, and the stamp will form a unique and interesting souvenir, besides affording the benevolent an opportunity of assisting the sick poor at the smallest inconvenience to themselves and at the maximum of benefit to the recipients, as all the money derived from the sale of the stamps will go to the fund.

The Indian Famine Fund amounts up to date to £497,000.

The annual meeting of the Children's Fresh Air Mission will be held in the Governors' Room of the Charterhouse on Friday, April 30th, at four o'clock.

Former house surgeons, dressers, and clerks of Lord Lister at Glasgow, Edinburgh, and London will give a banquet in honour of their former chief at the Café Royal on May 28th.

Mr. G. Stoker asks us to state that demonstrations are given on Tuesdays, Thursdays, and Saturdays at half-past four at the Oxygen Home, 2, Fitzroy Square, W.

The annual general meeting of the Society for Teaching the Blind, Swiss Cottage, will be held in the Concert Hall at the institution on Saturday, May 1st, at three p.m., the Lord Bishop of Stepney in the chair.

The opening of the Blackwall Tunnel by the Prince and Princess of Wales is fixed for Saturday, May 12th. The proceeds from the letting of seats on the stand which is to be erected for spectators will be devoted to the Seamen's Hospital, Greenwich.

The Hon. Thos. Bayard, the retiring Ambassador of the United States, will lay the foundation-stone of the new Home for Men presented by Mr. Passmore Edwards to the National Society for the Employment of Epileptics at the Colony, Chalfont St. Peter, Bucks, on Thursday, May 6th, at three o'clock. Mrs. Bayard will open the Home for Women at the Colony, also the gift of Mr. Edwards, on the same day.

On Monday the Japanese cruiser *Fuji* was inspected by a large number of visitors, the proceeds from the admission fees being given to the Seamen's Hospital. The *Fuji* is a fine armoured war ship of the class of the *Majestic* of our navy, now preparing for sea at Tilbury. A Japanese crew is already installed, and ready to work the ship to Japan when she is completed. The *Fuji* is a flagship, and the admiral's quarters are handsome and spacious. The wants of the sick are provided for in a most excellent dispensary which would put many a hospital dispensary to shame. The sick bay is not so spacious, and the berths are very closely packed together.

After some litigation the bequests of £9,000 by Mr. William Waller, of Gravesend, have been awarded by the Lord Mayor. Mr. Waller's life was saved by seamen when, as a youth, he was shipwrecked off the West Indies. His bequest was entirely in favour of seamen, and, besides the following hospitals which benefited, the Hospital at Gravesend received £3,000; to Greenwich Hospital, £2,000; the Seamen's Hospital Society (late "Dreadnought"), £3,000; Guy's Hospital (to endow two beds for seamen), £2,000; Poplar Hospital for Accidents, £1,000; the Royal Alfred Aged Merchant Seamen's Institution, £500; Merchant Seamen's Orphan Asylum, £200; Shipwrecked Fishermen and Mariners' Society, £105; Ramsgate Seamen's Infirmary, £105; and the Passmore Edwards District Cottage Hospital at Tilbury, £105.

